# Opportunistic Screening for Low Bone Density Using Automated Vertebral Trabecular CT Attenuation from Low-Dose CT Acquired During FDG PET/CT: A Single-Center Retrospective Study

**DOI:** 10.3390/tomography12060089

**Published:** 2026-06-17

**Authors:** Hyun-Kyeong Yuk, Sung-Hoon Oh, Do-Hoon Kim

**Affiliations:** 1 Department of Radiology, Daejeon Eulji Medical Center, Daejeon 35233, Republic of Korea; 20251163@eulji.ac.kr (H.-K.Y.); ohsh4803@eulji.ac.kr (S.-H.O.); 2 Department of Nuclear Medicine, Daejeon Eulji Medical Center, Eulji University School of Medicine, Daejeon 35233, Republic of Korea

**Keywords:** opportunistic screening, positron emission tomography/computed tomography, bone mineral density, deep learning, automated segmentation

## Abstract

Objectives: To evaluate the diagnostic performance of automated vertebral trabecular Hounsfield unit (HU) measurements derived from routine fluorodeoxyglucose positron emission tomography/computed tomography (FDG PET/CT) for identifying low bone density. Methods: This retrospective study included 131 consecutive women (mean age, 53.5 ± 9.6 years) undergoing health screening with FDG PET/CT and dual-energy X-ray absorptiometry (DXA) between January 2020 and December 2024. A deep learning-based model (TotalSegmentator) automatically segmented the lumbar vertebrae (L1–L4). HU-based metrics in trabecular regions were calculated, and their correlations with DXA-derived bone mineral density (BMD) were assessed. Diagnostic performance was evaluated using receiver operating characteristic analysis. A multivariable logistic regression model incorporating mean HU, age, and body mass index was developed and internally validated using bootstrap resampling. Results: According to WHO criteria, 47 of 131 participants (35.9%) had low bone density. Mean HU demonstrated strong diagnostic performance (area under the curve [95% confidence interval]: L1, 0.861 [0.800–0.923]; L2, 0.852 [0.788–0.915]; L3, 0.861 [0.800–0.921]; L4, 0.845 [0.781–0.909]). L1 mean HU provided the most balanced performance (sensitivity, 0.851; specificity, 0.750); L3 mean HU was slightly inferior. L1 mean HU was strongly correlated with BMD (r = 0.821, *p* < 0.001). In multivariable analysis, mean HU independently predicted low bone density (odds ratio: 0.949, *p* < 0.001). The model achieved an accuracy of 0.786 and demonstrated favorable calibration performance. Conclusions: The automated assessment of vertebral trabecular HU from routine FDG PET/CT provides a reliable and highly efficient method for screening low bone density without additional radiation exposure or cost.

## Simple Summary

Low bone density and osteoporosis are often underdiagnosed because dedicated bone density examinations are not routinely performed. This study developed an automated method to assess bone density using low-dose computed tomography images obtained during routine positron emission tomography/computed tomography examinations. The proposed approach showed good diagnostic performance and strong correlation with standard bone density measurements, without requiring additional radiation exposure or cost. Because positron emission tomography/computed tomography is widely used in clinical practice, this method may support large-scale opportunistic screening and improve the early identification of individuals at risk of osteoporosis.

## 1. Introduction

Osteoporosis is a major global health concern characterized by reduced bone strength and an increased risk of fragility fractures, resulting in substantial morbidity, mortality, and healthcare burden [1]. Early identification of individuals with low bone density, including those with osteopenia and osteoporosis, is critical for timely intervention and prevention of adverse clinical outcomes [2]. Dual-energy X-ray absorptiometry (DXA) remains the reference standard for assessing bone mineral density (BMD); however, its use in routine clinical practice is limited by underscreening, restricted accessibility, and the need for dedicated examinations [3,4]. Consequently, a substantial proportion of at-risk individuals remain undiagnosed until a fracture occurs.

In recent years, opportunistic screening using computed tomography (CT) has emerged as a promising alternative for assessing bone density. CT attenuation values, expressed in Hounsfield units (HU), have been shown to correlate strongly with BMD and can be derived from routine CT scans obtained for other clinical indications [5,6,7]. This approach enables the extraction of clinically relevant information without additional radiation exposure or cost. Several studies have demonstrated that vertebral HU measurements, particularly those from the lumbar vertebrae, can detect osteoporosis with good diagnostic accuracy [8,9,10,11]. Furthermore, recent large-scale studies and systematic reviews have highlighted the clinical potential of opportunistic CT screening and emphasized the need for standardized methodologies and reproducible workflows [12,13,14].

F-18 fluorodeoxyglucose (FDG) positron emission tomography (PET)/CT is widely performed in oncologic imaging for diagnosis, staging, and treatment monitoring. The CT component of PET/CT, although typically acquired at a low dose and without contrast enhancement, is routinely available across large patient populations and represents a valuable yet underutilized resource for opportunistic bone density assessment. Moreover, patients undergoing PET/CT represent a clinically relevant population for screening, as individuals with cancer are at an increased risk of low bone density due to aging, systemic disease, and treatment-related effects. Although prior studies have suggested the feasibility of assessing bone density using PET/CT-derived CT data, standardized automated approaches remain limited [15,16,17].

The accurate evaluation of trabecular bone is essential, as it is metabolically more active and more sensitive to changes in bone density than cortical bone [18,19]. However, reliable isolation of the trabecular compartment is technically challenging, particularly in low-dose CT images. Conventional approaches often rely on manual or semiautomated region-of-interest (ROI) selection, which introduces variability and limits reproducibility [20]. Recent advances in deep learning-based segmentation, including nnU-Net and TotalSegmentator, have enabled the automated and standardized identification of anatomical structures with high accuracy, thereby improving the reproducibility and scalability of quantitative imaging analyses [21,22].

Therefore, this study aimed to develop and evaluate an automated and reproducible framework for the opportunistic assessment of vertebral trabecular bone density using low-dose CT images acquired during routine FDG PET/CT. We assessed the diagnostic performance of HU-based metrics for identifying low bone density, evaluated their correlation with DXA-derived BMD, and examined the performance and calibration of a multivariable prediction model incorporating imaging and clinical variables. By leveraging routinely acquired imaging data without additional radiation exposure or cost, this approach has the potential to enable scalable and clinically applicable screening for low bone density.

## 2. Materials and Methods

### 2.1. Participant Selection and Bone Mineral Density Measurement

This study initially included consecutive female participants who underwent health screening with F-18 FDG PET/CT and DXA between January 2020 and December 2024. PET/CT and DXA examinations were performed within 1 week of each other.

The predefined exclusion criteria were as follows: (1) the presence of internal fixation devices or a history of spinal surgery in the lumbar region that precluded reliable HU measurement; (2) poor image quality due to severe motion or reconstruction artifacts, rendering automated segmentation and HU extraction infeasible; and (3) unavailable or incomplete DXA records.

The participant selection process is summarized in Figure S1. Among the initially screened participants (*n* = 164), 23 participants were excluded because of unavailable or incomplete DXA records, and 10 participants younger than 40 years were additionally excluded.

No participants were excluded because of lumbar fixation devices, prior spinal surgery, severe image artifacts, segmentation failure, or unsuccessful HU extraction. The final analysis included 131 participants.

Reference BMD was measured using a Horizon C DXA scanner (Hologic Inc., Marlborough, MA, USA). Lumbar spine DXA measurements were available separately for each vertebral level (L1–L4) and were used for vertebral-level-matched analyses with the corresponding CT-derived HU measurements. None of the participants met standard vertebral exclusion criteria for lumbar spine DXA interpretation, including severe vertebral deformity, metallic instrumentation, or other conditions affecting measurement reliability. According to the World Health Organization (WHO) classification based on DXA T-scores, the cohort consisted of 47 individuals with low bone density (osteopenia or osteoporosis) and 84 individuals with normal bone density.

### 2.2. CT Acquisition and Image Processing

Low-dose CT images acquired as part of FDG PET/CT examinations were used for analysis. All scans were performed using a Discovery 690 PET/CT scanner (GE Healthcare, Chicago, IL, USA) with a standardized imaging protocol and without intravenous contrast enhancement. CT acquisition parameters included a tube voltage of 120 kVp, automated tube current modulation (approximately 30–80 mAs), and a slice thickness of 3.75 mm. These parameters were consistent across all participants to ensure comparability of HU measurements.

### 2.3. Automated Vertebral Segmentation and Trabecular ROI Extraction

**Figure 1:**
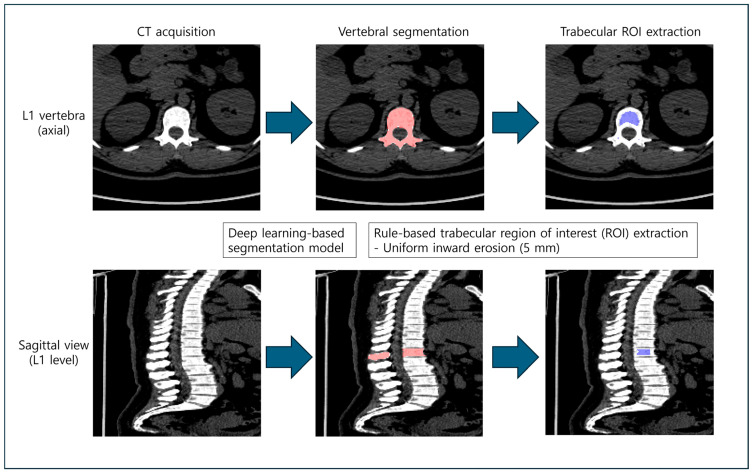
Automated workflow for the opportunistic assessment of vertebral trabecular bone density using CT images obtained during routine FDG PET/CT. Representative axial (**top** row) and sagittal (**bottom** row) images at the L1 vertebral level are shown. First column: Original CT images acquired as part of routine FDG PET/CT (bone window, −200 to 1200 HU). Second column: Automated vertebral segmentation generated using a deep learning-based model. Third column: Extraction of the trabecular ROI using a Euclidean distance transform-based inward erosion approach. The segmented vertebral body mask was uniformly eroded inward by 5.0 mm in physical space to minimize the influence of the cortical shell and vertebral endplates while preserving the central trabecular compartment. Sagittal images provide anatomical context and illustrate the spatial localization of the ROI within the L1 vertebral body. CT, computed tomography; FDG, fluorodeoxyglucose; HU, Hounsfield unit; PET, positron emission tomography; ROI, region of interest.

A deep learning-based whole-body segmentation framework, TotalSegmentator (version 2.9.0; University Hospital Basel, Basel, Switzerland), based on the nnU-Net architecture, was used to automatically segment the lumbar vertebrae (L1–L4). CT images were processed using the default preprocessing workflow implemented in TotalSegmentator. The publicly available pretrained model distributed with TotalSegmentator was used without additional retraining or fine-tuning. From each segmented vertebra, a trabecular ROI was extracted using a rule-based approach to minimize the influence of cortical bone and endplate effects (Figure 1). Specifically, the segmented vertebral mask was uniformly eroded inward by 5.0 mm in physical space using a Euclidean distance transform-based method to reduce the influence of the cortical shell and vertebral endplates while preserving the central trabecular compartment.

All segmentation and image processing procedures were performed on a workstation equipped with an NVIDIA RTX 5080 GPU. Although the pipeline was automated, all outputs underwent visual quality control by an experienced nuclear medicine physician with 10 years of experience to confirm anatomical plausibility and the absence of major segmentation errors. No manual correction was required in the final study cohort, and no separate vertebral-level exclusion or manual vertebral selection was performed after automated processing and visual quality control. In addition, the final cohort did not include vertebrae with severe focal abnormalities that precluded reliable quantitative HU analysis.

Because this study did not aim to develop or validate a segmentation algorithm, segmentation performance was not quantitatively evaluated (e.g., Dice similarity coefficient or Hausdorff distance). Instead, all segmentation outputs underwent visual quality control as described above.

### 2.4. HU Measurement

Within each vertebral trabecular ROI, HU-based metrics were calculated independently for each lumbar vertebral level (L1–L4), including the mean, median, and percentile values. Diagnostic analyses were performed separately for each vertebral level rather than using averaged multilevel HU values. Among these variables, L1 mean HU was selected as the primary variable for multivariable modeling because it demonstrated the most balanced diagnostic performance.

For vertebral-level analyses, CT-derived HU measurements from each lumbar vertebra (L1–L4) were matched with the corresponding DXA measurements from the same vertebral level within each patient. Correlation, receiver operating characteristic (ROC) curve, and regression analyses were then performed separately for each vertebral level.

### 2.5. Statistical Analysis

Continuous variables were expressed as means ± standard deviations, and categorical variables as counts and percentages. Between-group differences were evaluated using the independent t-test for continuous variables and the chi-square test for categorical variables.

Diagnostic performance for identifying low bone density was assessed using ROC curve analysis. The area under the curve (AUC), sensitivity, specificity, and optimal cutoff values (determined using the Youden index) were calculated.

The correlation between HU and DXA-derived BMD was assessed using Pearson’s correlation coefficient.

A multivariable logistic regression model incorporating the mean HU, age, and body mass index (BMI) was constructed to predict low bone density. Model performance was evaluated using classification metrics, including accuracy, sensitivity, and specificity.

Internal validation was performed using bootstrap resampling (1000 iterations). Calibration performance was assessed using calibration plots and mean absolute error.

All statistical analyses were performed using R v4.0.5 (R Foundation for Statistical Computing, Vienna, Austria).

## 3. Results

### 3.1. Study Population

A total of 131 participants were included, of whom 47 (35.9%) were classified as having low bone density (osteopenia or osteoporosis) and 84 (64.1%) as having normal bone density, based on DXA measurements (Table 1).

### 3.2. Diagnostic Performance of CT Attenuation

**Figure 2:**
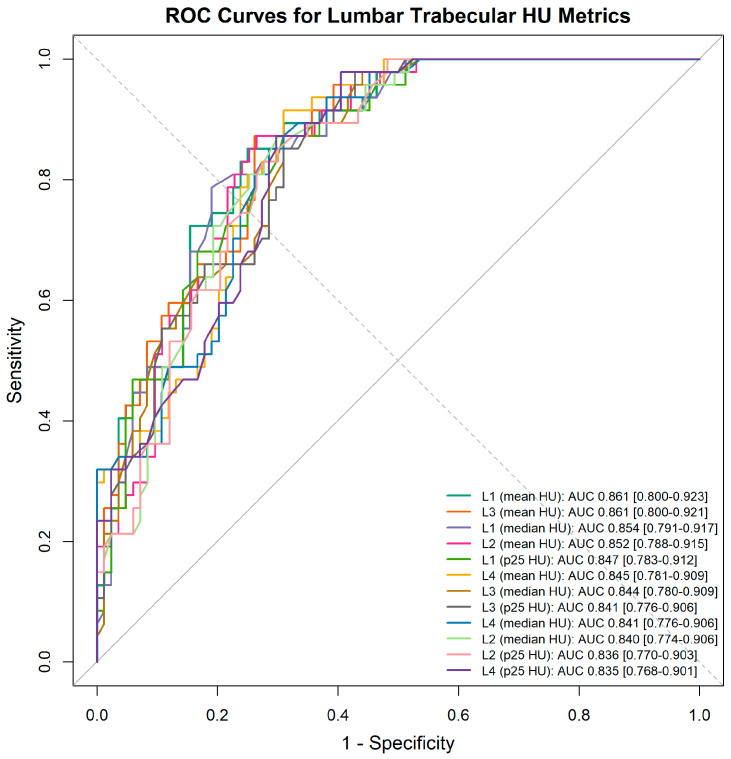
ROC curves of computed tomography attenuation-based metrics for identifying low bone density. ROC curves demonstrate the diagnostic performance of HU-derived parameters from the lumbar vertebrae (L1–L4) in differentiating low bone density (osteopenia and osteoporosis) from normal bone density. Among the evaluated metrics, the mean HU shows the highest and most consistent performance across all vertebral levels. The AUC values for the mean HU are 0.861 for L1, 0.852 for L2, 0.861 for L3, and 0.845 for L4. Overall, L1 mean HU provides the most balanced diagnostic performance, whereas L3 shows an equivalent AUC with a slightly higher sensitivity but lower specificity. The diagonal line indicates the line of no discrimination (AUC = 0.5). AUC, area under the curve; ROC, receiver operating characteristic.

ROC curve analysis demonstrated that HU-based metrics derived from the lumbar vertebrae exhibited good diagnostic performance for identifying low bone density (Figure 2). Among these metrics, mean HU consistently demonstrated the highest performance across vertebral levels.

The AUC values for mean HU were 0.861 for L1, 0.852 for L2, 0.861 for L3, and 0.845 for L4 (Table 2). L1 mean HU provided the most balanced diagnostic performance, whereas L3 demonstrated a comparable AUC, with slightly higher sensitivity but lower specificity.

### 3.3. Correlation Between HU and BMD

**Figure 3:**
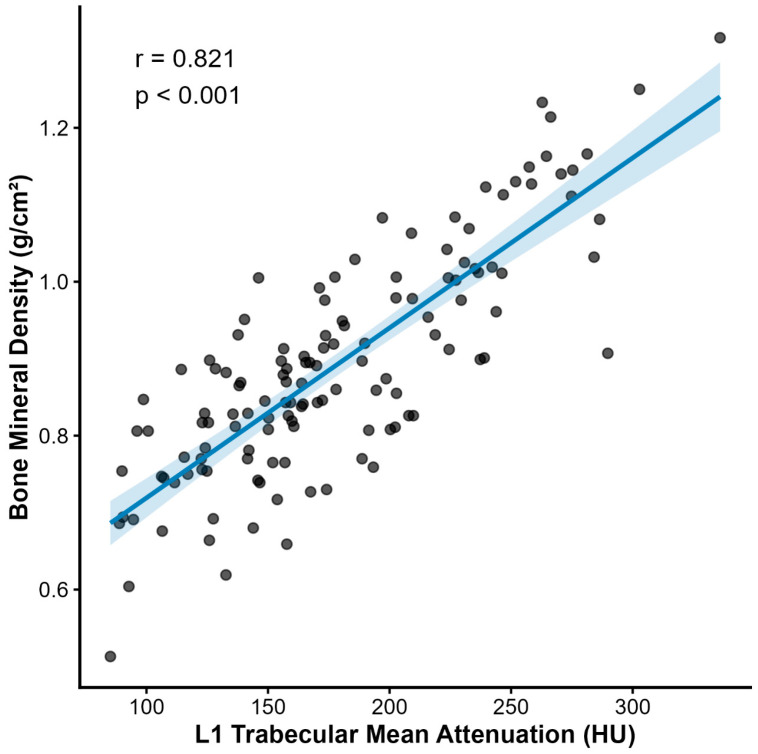
Relationship between L1 vertebral trabecular attenuation and BMD. Scatter plot showing the association between mean trabecular attenuation (HU) of the L1 vertebral body and BMD (g/cm^2^) measured using dual-energy X-ray absorptiometry. The solid line represents the linear regression fit, and the shaded area indicates the 95% CI. A strong positive correlation can be observed between HU and BMD.

A strong positive correlation was observed between the trabecular mean HU and DXA-derived BMD at the L1 vertebral level (Figure 3, Table 3). Higher HU values were associated with higher BMD, supporting the validity of CT attenuation as a surrogate marker of bone density.

### 3.4. Multivariable Logistic Regression Analysis

In the multivariable logistic regression analysis, mean HU remained a significant independent predictor of low bone density after adjustment for age and BMI (Table 3). Higher HU values were associated with a lower probability of low bone density.

### 3.5. Classification Performance

The logistic regression model demonstrated good classification performance, with an accuracy of 0.786, a sensitivity of 0.851, and a specificity of 0.750 (Table 3). The model achieved high sensitivity for detecting low bone density while maintaining acceptable specificity, indicating its potential utility as a screening tool.

### 3.6. Internal Validation and Calibration

**Figure 4:**
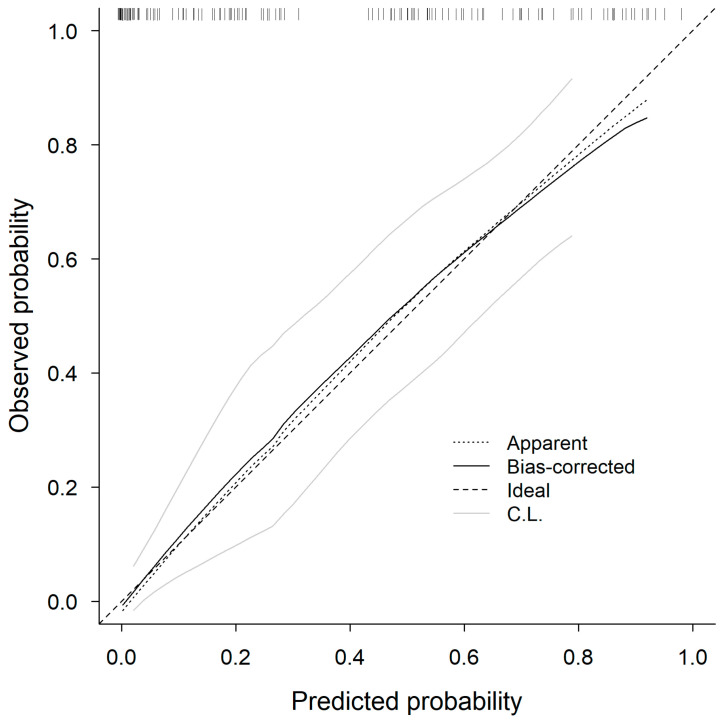
Calibration plot of the multivariable model for low bone density. The plot shows the apparent and bootstrap bias-corrected calibrations of the logistic regression model incorporating L1 trabecular mean attenuation, age, and BMI. The diagonal line represents ideal calibration, and the light gray lines, labeled as C.L. in the figure, indicate the 95% CI. The model showed a calibration intercept of 0.000 (95% CI, −0.477 to 0.473) and a calibration slope of 1.000 (95% CI, 0.672 to 1.412). Although the point estimates are close to the ideal values, the CIs indicate uncertainty in the calibration estimates. C.L., confidence limits.

Bootstrap-based internal validation demonstrated stable model performance with minimal optimism. The calibration plot showed good agreement between predicted and observed probabilities (Figure 4). The model demonstrated a mean absolute error of approximately 0.019, and the calibration curve closely approximated the ideal line. The calibration intercept and slope were 0.000 (95% confidence interval, −0.477 to 0.473) and 1.000 (95% CI, 0.672 to 1.412), respectively.

Overall, these findings suggest that the model provides reasonably reliable risk estimation with acceptable calibration performance. However, the calibration estimates should be interpreted with caution, given the width of the confidence intervals.

### 3.7. Subgroup Analysis in Women Aged ≥ 50 Years

A subgroup analysis was performed in women aged 50 years or older to assess the potential influence of menopausal status. The age distribution of the study population and the subgroup analysis results are summarized in Supplementary Table S1. Among the 83 women aged ≥ 50 years, 40 were classified as having low bone density and 43 as having normal bone density. L1 mean HU remained strongly correlated with DXA-derived BMD (r = 0.753, *p* < 0.001). In multivariable logistic regression analysis, mean HU remained an independent predictor of low bone density (OR = 0.957, 95% CI 0.933–0.976, *p* < 0.001), whereas age and BMI were not statistically significant predictors. Diagnostic performance remained acceptable, with an accuracy of 0.747, sensitivity of 0.800, specificity of 0.698, and balanced accuracy of 0.749.

## 4. Discussion

In this study, we demonstrated that vertebral trabecular attenuation derived from low-dose CT images acquired during routine FDG PET/CT enables the reliable identification of individuals with low bone density. The proposed automated framework showed strong diagnostic performance across lumbar vertebral levels, with an AUC of up to 0.861, and trabecular attenuation at the L1 level exhibited a strong correlation with DXA-derived BMD (r = 0.821). In addition, the multivariable model demonstrated good classification performance and generally favorable calibration, with minimal optimism observed in bootstrap validation. These findings support the feasibility of integrating opportunistic bone density assessment into routine PET/CT workflows without additional radiation exposure or cost.

The diagnostic performance observed in this study is consistent with the findings of prior CT-based opportunistic screening studies, which have reported AUC values typically ranging from approximately 0.80 to 0.90 for identifying osteoporosis using vertebral HU measurements [6,7,8,9,10,11]. Our results extend these findings to low-dose CT images acquired as part of PET/CT examinations, suggesting that reduced-dose, non-contrast CT images remain sufficiently informative for quantitative bone assessment. This is clinically relevant because PET/CT examinations are widely performed and generate large volumes of imaging data that can be leveraged for opportunistic screening.

Among the evaluated vertebral levels, L1 demonstrated the most balanced diagnostic performance. Although L3 showed an equivalent AUC with slightly higher sensitivity, this was achieved at the expense of lower specificity. The preferential use of L1 offers practical advantages, as L1 is more consistently included within the PET/CT field of view and is less affected by degenerative changes than lower lumbar levels. These characteristics may improve reproducibility and facilitate standardization across institutions, which is essential for clinical implementation.

A key strength of this study is the automated and reproducible extraction of trabecular bone regions. By combining deep learning-based vertebral segmentation with standardized Euclidean distance transform-based inward erosion of the vertebral mask in physical space, the influence of cortical bone and vertebral endplates was minimized while preserving the central trabecular compartment. This automated framework effectively overcomes the limitations of prior studies that relied on manual or semiautomated ROI placement, which is inherently subject to interobserver variability [20]. The use of state-of-the-art segmentation frameworks, such as nnU-Net and TotalSegmentator, further supports the robustness and scalability of the proposed pipeline [21,22].

The multivariable logistic regression model demonstrated that mean HU was an independent predictor of low bone density after adjustment for clinical variables. By contrast, BMI was not a significant predictor in the multivariable model, suggesting that CT-derived trabecular attenuation may provide more direct and sensitive information regarding bone quality than general anthropometric measures. Notably, the model demonstrated generally good calibration. Although the calibration intercept (0.000) and slope (1.000) were close to their ideal values, the associated confidence intervals were relatively wide, indicating uncertainty in these estimates. Therefore, the calibration results should be interpreted cautiously and should be confirmed through external validation in larger and more diverse cohorts. Calibration is increasingly recognized as a critical component of predictive modeling because it reflects the agreement between predicted and observed probabilities and determines clinical utility [23]. The development and reporting of the prediction model were aligned with established methodological frameworks for clinical prediction models, including the Transparent Reporting of a multivariable prediction model for Individual Prognosis Or Diagnosis (TRIPOD) statement [24]. The calibration results observed in this study support the potential utility of the model for risk stratification. However, confirmation in larger external cohorts is required before broader clinical implementation.

This study has several important clinical implications. The opportunistic assessment of bone density using PET/CT-derived CT images enables the identification of at-risk individuals without additional imaging, cost, or radiation exposure. Patients undergoing PET/CT—particularly those with malignancy—represent a population at an increased risk of bone loss, making them an appropriate target for opportunistic screening. Furthermore, the highly automated nature of the proposed framework supports seamless integration into clinical workflows, allowing bone density information to be generated alongside routine imaging reports.

The study population was limited to female participants to ensure a homogeneous cohort. Given that body composition, bone mineral distribution, and patterns of bone loss differ significantly between sexes [25], the relationship between HU and BMD, as well as optimal diagnostic cutoff values, may be confounded by sex-specific physiological differences [26]. By focusing exclusively on women, we aimed to improve the precision of our estimates and establish reliable diagnostic thresholds for this high-risk population.

Recent studies have increasingly explored automated artificial intelligence-based approaches for opportunistic osteoporosis screening using routine CT examinations. In 2021, Löffler et al. demonstrated automated opportunistic osteoporosis screening using routine CT examinations and reported improved identification of patients with prevalent vertebral fractures [27]. Building upon these early efforts, more recent deep learning frameworks have demonstrated excellent performance for automated vertebral localization, segmentation, and bone density assessment across various CT protocols and scanner vendors. Oh et al. reported that deep learning-based automated quantitative CT measurements achieved strong agreement with conventional quantitative CT and showed promising diagnostic performance for osteoporosis screening using routine chest, abdominal, and lumbar CT examinations [28]. In 2024, Park et al. demonstrated the robustness of automated BMD assessment across multiple CT protocols and scanner vendors, supporting the feasibility of large-scale opportunistic screening in real-world clinical settings [29]. Furthermore, multicenter validation studies have shown that deep learning-based CT attenuation analysis can maintain acceptable diagnostic performance across institutions, highlighting the potential for broader clinical implementation [30].

Compared with these CT-based studies, our work has several distinctive features. First, the proposed framework was specifically developed for low-dose CT images acquired as part of routine FDG PET/CT examinations. Because FDG PET/CT is widely performed for health screening and oncologic evaluation, the ability to derive bone density information from existing examinations may provide additional clinical value without additional imaging, radiation exposure, or workflow modification. Second, vertebral segmentation was performed automatically using a deep learning-based model, followed by standardized extraction of the trabecular compartment using a rule-based morphological approach designed to minimize the influence of cortical bone. Third, quantitative HU measurements were obtained without manual vertebral selection, manual ROI placement, or vertebral-level exclusion in the final study cohort. These characteristics enable a practical end-to-end workflow that may facilitate opportunistic osteoporosis screening directly from routine PET/CT examinations.

Previous PET/CT-based studies have demonstrated correlations between vertebral CT attenuation and BMD; however, many relied on manual or semi-automated vertebral selection and HU measurements. Unlike these previous PET/CT-based approaches, our framework enables automated vertebral segmentation, standardized trabecular ROI extraction, and quantitative HU assessment within a unified workflow, thereby minimizing operator dependency and improving reproducibility. These characteristics may facilitate future large-scale implementation.

### Study Limitations

This study has some limitations. First, this was a retrospective, single-center study, which may limit the generalizability of the findings; therefore, external validation across multiple institutions and scanner types is required. In addition, although no cases were excluded because of segmentation failure or image artifacts in the present cohort, the study population consisted of relatively standardized health screening examinations from a single center. Therefore, further validation in more heterogeneous real-world datasets is warranted to confirm the robustness and generalizability of the proposed framework. Although bootstrap-based internal validation demonstrated stable model performance and favorable calibration, internal validation cannot fully assess generalizability to independent populations. All examinations in the present study were acquired using a single PET/CT scanner (Discovery 690, GE Healthcare) with a standardized low-dose CT protocol. Therefore, the observed diagnostic performance and calibration may not directly generalize to other institutions, scanner vendors, or acquisition environments. Technical factors, including scanner calibration, tube voltage, reconstruction kernel, iterative reconstruction algorithms, slice thickness, and dose settings, may influence absolute HU measurements and potentially affect diagnostic cutoff values. Future multicenter studies involving diverse PET/CT systems and acquisition protocols will be necessary to evaluate the robustness of the proposed framework, establish harmonized HU thresholds, and confirm its clinical applicability across different imaging environments. Second, although DXA was used as the reference standard, differences in anatomical measurement sites and temporal intervals may have introduced bias. Third, although the automated segmentation approach enhances reproducibility, segmentation errors cannot be entirely excluded. In addition, although a fixed erosion distance improves standardization and reproducibility, vertebral size variability may influence the proportion of retained trabecular bone, and adaptive anatomy-aware erosion strategies should be explored in future studies. Fourth, we evaluated diagnostic performance based on individual vertebral levels. Future studies are warranted to investigate whether combining multiple vertebral levels (such as L1–L2 or L1–L4) improves diagnostic performance and robustness. Fifth, the study cohort consisted exclusively of women aged 40 years or older undergoing health-screening FDG PET/CT examinations, which may limit the generalizability of the findings to broader populations, including men and younger individuals. Menopausal status was not consistently available because of the retrospective study design and therefore could not be included in the analysis. In addition, the term “low bone density” in the present study was defined according to DXA-derived T-score criteria and was used as an analytical classification rather than a formal clinical diagnosis. This distinction is particularly important in younger and potentially premenopausal women, for whom interpretation based solely on T-scores may not fully reflect current clinical diagnostic recommendations. Therefore, the results should be interpreted as reflecting reduced bone density relative to the reference population rather than a definitive diagnosis of osteoporosis. Given the known effects of menopause and aging on bone metabolism, future studies incorporating menopausal status and age-stratified analyses are warranted. Sixth, prospective studies are needed to evaluate the ability of this approach to predict clinically relevant outcomes, such as fracture risk. Finally, although bootstrap-based internal validation suggested favorable calibration, the confidence intervals for the calibration estimates remained relatively wide because of the modest sample size. Therefore, the calibration performance should be interpreted cautiously and requires confirmation in independent external datasets.

## 5. Conclusions

Automated assessment of vertebral trabecular attenuation using low-dose CT from FDG PET/CT represents a reliable, scalable, and clinically applicable approach for identifying individuals with low bone density. This method demonstrated strong diagnostic performance and promising calibration performance for opportunistic screening, supporting its potential integration into routine clinical practice.

## Figures and Tables

**Table 1:** Baseline characteristics of the study population.

Variable	Low Bone Density (*n* = 47)	Normal Bone Density (*n* = 84)	*p*-Value
Age (years)	57.74 ± 9.20	51.08 ± 9.14	<0.001
Height (cm)	157.41 ± 4.59	160.32 ± 5.43	0.002
Weight (kg)	55.78 ± 7.79	60.38 ± 9.96	0.008
BMC (g)	10.18 ± 1.46	13.65 ± 2.27	<0.001
BMD (g/cm^2^)	0.76 ± 0.07	0.97 ± 0.12	<0.001
T-score	−1.56 ± 0.66	0.32 ± 1.06	<0.001
PR (%)	81.26 ± 7.94	103.87 ± 12.66	<0.001
AM (%)	94.32 ± 10.32	113.02 ± 12.27	<0.001
L1 mean HU	136.77 ± 29.90	201.17 ± 50.91	<0.001
L2 mean HU	137.29 ± 32.43	203.18 ± 53.08	<0.001
L3 mean HU	138.09 ± 33.55	208.39 ± 54.02	<0.001
L4 mean HU	143.94 ± 34.22	214.93 ± 56.44	<0.001

Values are presented as mean ± standard deviation. Low bone density includes osteopenia and osteoporosis. AM, age-matched percentage; BMC, bone mineral content; BMD, bone mineral density; PR, percentage of young adult reference.

**Table 2:** Diagnostic performance of CT attenuation (mean HU) for identifying low bone density.

Variable	AUC	Cutoff (HU)	Sensitivity	Specificity
L1 mean HU	0.861	164.15	0.851	0.75
L2 mean HU	0.852	167.88	0.872	0.735
L3 mean HU	0.861	169.85	0.872	0.738
L4 mean HU	0.845	184.60	0.915	0.690

Optimal cutoff values were determined using the Youden index. Low bone density includes osteopenia and osteoporosis.

**Table 3:** Correlation, multivariable logistic regression, and classification performance of L1 CT attenuation. (**A**) Correlation between CT attenuation and BMD. (**B**) Multivariable logistic regression analysis. (**C**) Classification performance.

(**A**)			
**Variable**	**Correlation Coefficient (r)**		***p*-Value**
Mean HU	0.821		<0.001
Median HU	0.809		<0.001
p25 HU	0.808		<0.001
p75 HU	0.815		<0.001
p90 HU	0.817		<0.001
(**B**)			
**Variable**	**OR**	**95% CI**	***p*-Value**
Mean HU	0.949	0.928–0.967	<0.001
Age	0.944	0.878–1.011	0.105
BMI	0.885	0.749–1.022	0.121
(**C**)			
**Metric**			**Value**
Accuracy			0.786
Sensitivity			0.851
Specificity			0.75
Positive predictive value			0.656
Negative predictive value			0.9
Balanced accuracy			0.801

Low bone density includes osteopenia and osteoporosis. BMI, body mass index; CI, confidence interval; OR, odds ratio.

## Data Availability

The data used in this study are not publicly available because they contain sensitive patient information and are subject to institutional data protection policies.

## Supplementary Materials

The following supporting information can be downloaded at: https://www.mdpi.com/article/10.3390/tomography12060089/s1, Figure S1: Flowchart of participant selection for the study cohort; Table S1: Age distribution and subgroup analysis restricted to women aged 50 years or older.

## Abbreviations

The following abbreviations are used in this manuscript:

AM	Age-matched percentage
AUC	Area under the curve
BMC	Bone mineral content
BMD	Bone mineral density
BMI	Body mass index
CI	Confidence interval
C.L.	Confidence limits
CT	Computed tomography
DXA	Dual-energy X-ray absorptiometry
FDG	Fluorodeoxyglucose
HU	Hounsfield unit
OR	Odds ratio
PET	Positron emission tomography
PR	Percentage of young adult reference
ROC	Receiver operating characteristic
ROI	Region of interest
TRIPOD	Transparent Reporting of a multivariable prediction model for Individual Prognosis Or Diagnosis
WHO	World Health Organization
