# Supplementary material for: Opportunistic Screening for Low Bone Density Using Automated Vertebral Trabecular CT Attenuation from Low-Dose CT Acquired During FDG PET/CT: A Single-Center Retrospective Study
Source: Tomography. 2026 Jun 17;12(6):89. doi: 10.3390/tomography12060089 (PMC13307040; doi:10.3390/tomography12060089)
Supplement: Supplementary file 1 [file tomography-12-00089-s001.zip › tomography-4333156-supplementary.pdf]

tomography

## Supplementary Material

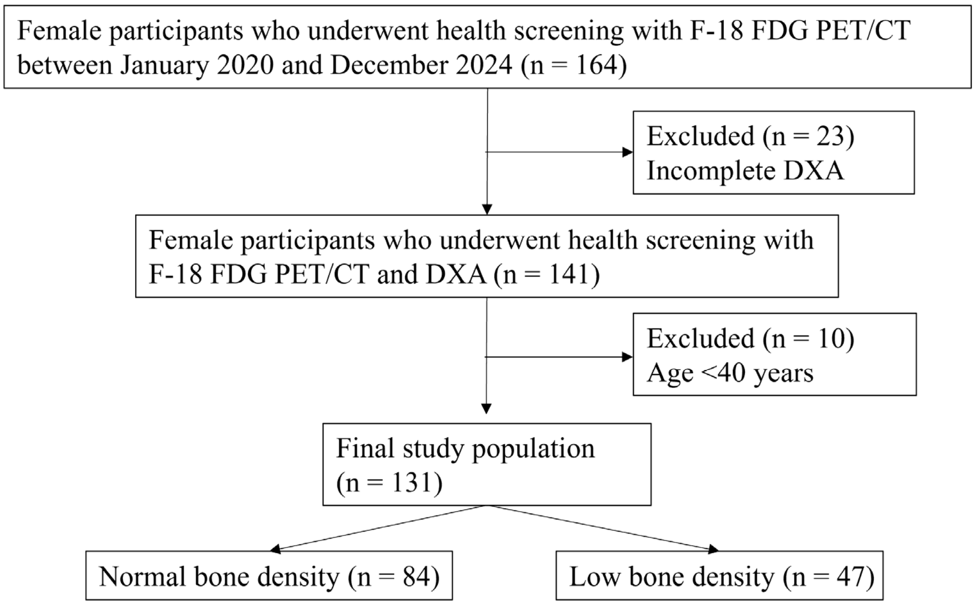


**Figure S1.** Flowchart of participant selection for the study cohort. Among female participants who underwent health-screening FDG PET/CT between January 2020 and December 2024, participants with unavailable or incomplete DXA records and those younger than 40 years were excluded. No participants were excluded because of segmentation failure, severe image artifacts, lumbar fixation devices, prior spinal surgery, or unsuccessful HU extraction. DXA, dual-energy X-ray absorptiometry; FDG, fluorodeoxyglucose; HU, Hounsfield unit; PET, positron emission tomography.

**Table S1.** Age distribution and subgroup analysis restricted to women aged 50 years or older.

(A) Age distribution according to the WHO classification.

| Age group (years) | Normal bone density | Low bone density | Total |
|---|---|---|---|
| 40–49 | 41 | 7 | 48 |
| 50–59 | 26 | 21 | 47 |
| 60–69 | 14 | 13 | 27 |
| ≥70 | 3 | 6 | 9 |
| Total | 84 | 47 | 131 |

(B) Comparison of the primary study results between the overall cohort and women aged ≥50 years.

| Variable | Overall cohort (n=131) | Age ≥50 years (n=83) |
|---|---|---|
| Mean HU–BMD correlation (r) | 0.821 | 0.753 |
| Mean HU, OR [95% CI] | 0.949 [0.928–0.967] | 0.957 [0.933–0.976] |
| Accuracy | 0.786 | 0.747 |
| Sensitivity | 0.851 | 0.800 |
| Specificity | 0.750 | 0.698 |
| Balanced accuracy | 0.801 | 0.749 |

The correlation coefficient (r) represents the Pearson correlation between L1 mean HU and DXA-derived BMD. ORs were derived from multivariable logistic regression models adjusted for age and body mass index. Low bone density includes osteopenia and osteoporosis. BMD, bone mineral density; CI, confidence interval; DXA, dual-energy X-ray absorptiometry; HU, Hounsfield unit; OR, odds ratio; WHO, World Health Organization.

https://doi.org/10.3390/tomography12060089
